# Quality of Life Assessment for Men With Gynecomastia in Saudi Arabia

**DOI:** 10.7759/cureus.30925

**Published:** 2022-10-31

**Authors:** Muna F Alnaim, Jawaher I Alraihan, Nouf M Al Rabiah, Basem Zogel, Samar M Alfaifi, Aishah N Azam, Abdulrahman O Baghdadi, Basem K Alhusaini

**Affiliations:** 1 Department of Surgery, King Faisal University, Al Hofuf, SAU; 2 Department of surgery, King Faisal University, Al Hofuf, SAU; 3 Medicine and Surgery, Jazan University, Jazan, SAU; 4 Department of Surgery, King Salman Bin Abdulaziz Medical Hospital, Al Madinah, SAU; 5 Department of Surgery, King Abdulaziz Hospital, Jeddah, SAU; 6 Department of Plastic and Reconstructive Surgery, King Fahad General Hospital, Al Madinah, SAU

**Keywords:** breast reduction, gynecomastia, questionnaire study, kingdom of saudi arabia (ksa), mastectomy, quality of life (qol)

## Abstract

Background: Gynecomastia, defined as a benign proliferation of the breasts in males, is a common pathology of breasts among adolescent males. The root cause behind the condition is the hormonal imbalance between androgens and estrogens at the time of puberty. Different treatment options can be used; however, surgery is the preferred option. The quality of life (QoL) of affected Individuals is usually affected in all aspects.

Objectives: The aim of the study is to assess the QoL of male Saudi patients diagnosed with gynecomastia and their desire to undergo surgical treatment.

Methods: A cross-sectional study was conducted using an Arabic self-administered online questionnaire that targeted males in Saudi Arabia and was distributed throughout the kingdom.

Results: A total of 681 participants were involved in this study. Most of the participants were Saudi (n = 607; 90.6%) and married (n = 158; 79%). Approximately, half of the participants were between 18 and 29 years of age (n = 337; 49.5%), while 41.1% and 9.3% were between 30 and 49 years and more than 50 years old, respectively. About 29.5% of the participants were from the middle region, while 26.7% of them were from the northern region, followed by 19.5% of participants from the western region, and only 14.4% and 9.8% from the eastern or southern region. On analysis of different domains, there was no statistically significant difference between participants with gynecomastia and the control group in QoL.

Conclusions: Patients showed no statistically significant change in the QoL between those diagnosed with gynecomastia and those in the control group. Also, more than one-third of our patients did not want to undergo breast reduction surgery.

## Introduction

Gynecomastia is a common mammary anomaly in adolescent men. It is defined as a benign proliferation of glandular tissue of the breast in men [1]. Asymptomatic gynecomastia is quite prevalent and has a trimodal demographic breakdown, appearing in neonatal, adolescents, and in older age. Asymptomatic gynecomastia affects 60%-90% of newborns, 50%-60% of children and teenagers, and up to 70% of adult men aged 50-69 years [2-5]. It occurs due to the imbalance between the androgens and estrogens at the breast tissue level. This is a principal reason for the development of gynecomastia, and the use of numerous medications is an important factor [6,7]. An initial clinical examination must be conducted to rule out pseudogynecomastia or other breast pathologies. Mammography can distinguish true gynecomastia from a tumor that requires a biopsy. It is also shown to be reasonably accurate in discriminating between malignant and benign male breast conditions and can significantly reduce the need for biopsies [8].

If the patient's gynecomastia persists and is associated with pain or psychological suffering, pharmaceutical and surgical alternatives are recommended. Drug therapy is more likely to be useful if it is begun before fibrous tissue substitutes glandular tissue, whereas surgery can be done at any time [9]. The gold standard treatment for gynecomastia is surgery. The standard method used is subcutaneous mastectomy, which primarily involves resection of the breast tissues with a peri-areolar approach that can be accompanied by liposuction. However, if breast enlargement is solely attributable to abundant fatty tissue and there is no major glandular hypertrophy, liposuction alone may be sufficient [10]. In genuine gynecomastia surgeries, histopathological testing is advised since unanticipated histological findings such as spindle-cell hemangioendothelioma and papilloma can arise in 3% of patients [11]. Our study was directed at the Saudi male population who needed treatment for gynecomastia. The aim was to assess their quality of life (QoL) and compare it to the unaffected male population in order to explore the psychological impact of this problem in the context of Saudi Arabia.

## Materials and methods

This cross-sectional descriptive study was conducted from January 2022 to August 2022. A total of 681 respondents were recruited for the study using a simple random sampling technique and were from all regions of Saudi Arabia. The research was approved by the institutional review board of King Faisal University, Al Hofuf, Saudi Arabia, with project number EA000479.

Inclusion and exclusion criteria

The inclusion criterion was adult men, 18 years and above, living in Saudi Arabia. People living outside Saudi Arabia and females were excluded from the study.

Sample size calculation

The sample size was calculated using a 5% margin of error and a 95% confidence interval, assuming a 50% response rate on average for most of the questions, based on the average number of the adult population in the Saudi Arabia region being 35.84 million, according to General Authority for Statistic Kingdom of Saudi Arabia in 2022. The required sample for the study is 385 participants. A total number of 681 responses were recorded in the study.

Data collection and management

A self-administered survey was used with the items of the questionnaire fully constructed by an expert, and a pilot study of 20 participants was done. The pilot study participants were excluded from participating in the study. The validity was completed through reviewing by three field experts. Along with demographic questions, the participants answered 39 questions that evaluated the factors that led people to seek out breast reduction surgery and used the WHO brief quality of life assessment in Arabic to measure the QoL. After receiving approval from King Faisal University's institutional review board (approval number: EA000479), the survey was sent to all Saudi adults. After being approved, the subjects were asked to complete surveys via WhatsApp groups starting on March 1, 2022. Data collection, anonymity, and each subject's right to decline participation have all been explained to the participants.

Statistical analysis

The World Health Organization Quality of Life Brief Version (WHOQOL-BREF) questionnaire, which has 26 questions, was used to assess the impact on QoL. Twenty-four questions evaluate the four domains of physical health (seven items), psychological health (six items), social relationships (three items), and environment (eight items). Two questions evaluate the personal assessment of overall perception of QoL and satisfaction with health. Each question has a five-point scale. A better QoL is indicated by higher scores.

Data were entered into Microsoft Excel (Microsoft Corporation, Redmond, Washington) for coding and analyzed by Statistical Package for the Social Sciences (SPSS; IBM Corp., Armonk, NY) using the chi-square test and Z test. The student’s t-test (t) was used to detect the mean difference between the two groups. A p-value of less than 0.05 was considered statistically significant.

## Results

A total of 681 participants who fulfilled the inclusion criteria were involved in this study. As seen in Table 1, most of the participants were Saudi (n = 607; 90.6%) and married (n = 158; 79%). Approximately, half of the participants were between 18 and 29 years of age (n = 337; 49.5%), while 41.1% and 9.3% were between 30 and 49 years and more than 50 years old, respectively. About 29.5% of the participants were from the middle region, while 26.7% of them were from the northern region, followed by 19.5% of participants from the western region, and only 14.4% and 9.8% from the eastern or southern region. Study respondents showed various educational backgrounds with most of them having bachelor’s degrees (57.4%), followed by 28.2% of participants holding high-school degrees, and only 5% and 1.3% completed middle and primary school. In regard to employment status, more than one-third were students (34.1%), 17.5% of the participants had a desk job, 10.1% were unemployed, 10% and 9.3% were teachers and health professionals, respectively, and 19.1% reported to have other jobs at the time of conducting this study. About 61.4% of the participants had been diagnosed with gynecomastia (n = 418). Most of them do not plan on treatment (44.9%), 23.1% of them are searching for non-surgical options, 18.2% of participants are following up with a surgeon for surgery, and only 13.8% did a mastectomy.

**Table 1:** Sociodemographic characteristics of the participants (n = 681) SAR: Saudi Arabian Riyal.

Variables	n	%
Age (years)		
18-29	337	49.5%
30-49	280	41.1%
<50	63	9.3%
Nationality		
Saudi	607	90.6%
Non-Saudi	64	9.4%
Marital status		
Single	403	59.2%
Married	278	40.8%
Region		
Middle	201	29.5%
Western	133	19.5%
Northern	182	26.7%
Eastern	98	14.4%
Southern	67	9.8%
Education level		
Primary school	9	1.3%
Middle school	34	5%
High school	192	28.2%
Bachelor’s degree	391	57.4%
Illiterate	5	0.7%
Employment status		
Unemployed	69	10.1%
Student	232	34.1%
Teacher	68	10%
Desk job	119	17.5%
Healthcare professional	63	9.3%
Others	130	19.1%
Currency income (SAR)		
Less than 10,000	82	41%
10,000 to 20,000	61	30.5%
21,000-30,000	49	24.5%
More than 30,000	8	4%
Were you ever diagnosed with gynecomastia?		
Yes	418	61.4%
No	263	38.6%
What was the next action you have done?		
Did a mastectomy	94	13.8%
Currently following up with a surgeon for surgery	124	18.2%
Searching for non-surgical options	157	23.1%
I don’t plan on treating my gynecomastia	306	44.9%

The motivation for surgical and non-surgical breast reduction procedures is shown in Table 2. The most frequently reported motivators for considering the surgery were feeling embarrassed wearing some clothes (24.7%) and seeing a good result on a friend (18.1%). About 14.1% of participants thought that doing the surgery will help to restore confidence. Getting bullied or negative comments were the least reported motivator. More than one-third of the participants do not plan on doing the surgery. As for barriers, nearly 19.7% of participants were worried about the danger of surgery, 14.7% of them stated that gynecomastia does not bother them, while 11.5% were not having enough money to undergo the surgery. Long medical work, being too busy to do the surgery, and believing that there are no satisfying results were the least reported barriers (9.5%, 8.2%, and 8.1%, respectively).

**Table 2:** Motivating factor for seeking surgical and non-surgical breast reduction procedures

Variables	Responses	n	%
Why are you planning on doing the surgery?	I saw a good result on a friend	123	18.1%
	I feel embarrassed wearing some clothes	168	24.7%
	I am going for surgery because I got bullied/negative comments	40	5.9%
	Because it will help me restore my confidence	96	14.1%
	I don’t plan on treating my gynecomastia	251	36.9%
Why do you not want to undergo surgery?	Because it's dangerous	134	19.7%
	There are no satisfying results	55	8.1%
	Because of the long medical work	65	9.5%
	Because gynecomastia doesn’t bother me	100	14.7%
	Because I don’t have enough money	78	11.5%
	Because I am too busy to do the surgery	56	8.2%

As shown in Table 3, most of the participants in our study (about 61.4%) were diagnosed with gynecomastia, whereas approximately 38.6% were without gynecomastia. However, the age, region, and employment status were statistically significant between the two groups (p = 0.001, p = 0.001, and p = 0.019, respectively).

**Table 3:** Comparison between healthy participants and participants with gynecomastia ** represents a significant value in the chi-square test (considered when 0.05 or less). SAR: Saudi Arabian Riyal.

Factors	Participants with gynecomastia (n = 418)	Participants without gynecomastia (n = 263)	p-value**
Age group (years)			0.001**
18-29	138 (43.9%)	154 (58.6%)	
30-49	183 (43.9%)	97 (36.9%)	
<50	51 (12.2%)	12 (4.6%)	
Nationality			0.203
Saudi	374 (89.5%)	243 (92.4%)	
Non-Saudi	44 (10.5%)	20 (7.66%)	
Currency income (SAR)			0.058
Less than 10,000	227 (54.3%)	169 (64.3%)	
10,000 to 20,000	142 (34%)	65 (24.7)	
21,000-30,000	34 (8.1%)	19 (7.2)	
More than 30,000	15 (3.6%)	10 (3.8)	
Marital status			0.097
Married	237 (56.7%)	166 (63.1%)	
Not married	181 (43.3%)	97 (36.9%)	
Region			0.001**
Middle	105 (25.1%)	96 (36.5%)	
Western	78 (18.7%)	55 (20.9%)	
Northern	107 (25.6%)	75 (28.5%)	
Eastern	68 (16.3%)	30 (11.3%)	
Southern	60 (14.4%)	7 (2.7%)	
Education level			0.135
Primary school	3 (0.7%)	6 (2.3%)	
Middle school	23 (5.5%)	11 (4.2%)	
High school	116 (27.8%)	76 (8.9%)	
Bachelor’s degree	235 (56.2%)	156 (59.3%)	
Illiterate	3 (0.7%)	2 (0.8)	
Employment status			0.019**
Unemployed	39 (9.3%)	30 (11.4%)	
Student	130 (31.1%)	102 (38.8%)	
Teacher	52 (12.4%)	16 (6.1%)	
Desk job	76 (18.2%)	43 (16.3%)	
Healthcare professional	45 (10.8%)	18 (6.8%)	
Others	76 (18.2%)	54 (20.5%)	

As shown in Table 4, the mean scores for participants with gynecomastia and the control group in the physical health domain were M = 60.4, SD = 14 and M = 59.5, SD = 16.3, respectively; psychological health domain were M = 60.46, SD = 15 and M = 61.4, SD = 17.6, respectively; social relationship domain were M = 64.11, SD = 21.4 and M = 61.14, SD = 25.7, respectively; and the environmental domain were M = 664.3, SD = 18.9 and M = 65.8, SD = 19.8, respectively. On analysis of different domains, there was no statistically significant difference between the participants with gynecomastia and the control group in QoL.

**Table 4:** Effect of gynecomastia on WHOQOL-BREF domains ** represents a significant value in the t-test (considered when 0.05 or less). SD: Standard deviation; WHOQOL-BREF: World Health Organization Quality of Life Brief Version.

WHOQOL-BREF domains	Participants with gynecomastia (n = 418)		Participants without gynecomastia (n = 263)		p-value**
	Mean	SD	Mean	SD	
Physical health	60.4	14	59.5	16.3	0.454
Psychological	60.46	15	61.4	17.6	0.476
Social relationships	64.11	21.4	64.14	25.7	0.987
Environment	64.3	18.9	65.8	19.8	0.301

## Discussion

This study’s objective was to discover the motivating factors for seeking breast reduction surgery in patients diagnosed with gynecomastia in Saudi Arabia and to assess their QoL. This is one of the first studies in the literature to explore the motivating factors for seeking surgery for gynecomastia patients in Saudi, according to the author’s knowledge. Six hundred and eighty-one participants filled out the questionnaire and shared it via the online survey. The most influential factor was the conscious feeling of embarrassment while wearing clothes; this is consistent with the study of Kinsella et al. [12]. The study was conducted on 24 patients who were diagnosed with gynecomastia, and it showed higher levels of anxiety, depression, and social phobia among them. In addition, surgery was thought to be the main option to restore confidence in the respondents, and this corresponds to what Kasielska-Trojan et al. [13] found as a motivational factor in their study. The most significant barrier was the fear of undergoing the surgery. In Saudi Arabia, there is a lack of awareness exemplified by a study made by Al Jabr et al.; they reported an overall insufficient knowledge regarding gynecomastia in students [14]. The last significant barrier was financial; respondents reported the inability to bear the financial burden of surgery. This can be attributed to the fact that 31.1% of the respondents were students with no financial income.

The least influential barriers were long medical work, being too busy for surgery, and having the presumption that there are no satisfying results after surgery which Ridha et al. touched on in their research where 74 were sent a survey after undergoing breast reduction surgery for gynecomastia, and it showed a 62.5% satisfaction rate, from which it is evident that there is way more than expected dissatisfaction post-surgery [15]. Surprisingly, the least motivational factor in this study is the bullying and negative comments opposed to the study by Kinsella et al. [12], which showed significant limitations of social activity, especially due to peers' negative attitude toward young adults with gynecomastia. More than one-third of the respondents do not plan on undergoing surgery, which can be explained by the fact that the QoL assessment was statistically insignificant between the people diagnosed with gynecomastia and those who are not. The middle region was found to be statistically significant in the numbers of those diagnosed with gynecomastia, which is limited by the fact that there are no reports of gynecomastia prevalence in Saudi medical literature to support our study. Also, another limitation was the lack of research in the Middle East to report the prevalence of this condition and its psychological impact. Moreover, the potential use of a more sensitive tool to assess the QoL than the one used in this study could add more accuracy. Lastly, more participants should be recruited to get a truer representation of the Saudi population.

## Conclusions

This study assessed the patients' QoL after being diagnosed with gynecomastia and their desire to perform breast reduction surgery in Saudi Arabia. Most patients diagnosed with gynecomastia showed no statistically significant change in the QoL compared to the control. Our results demonstrate that there is a statistically significant correlation between the age, region, and employment status in the two groups (p = 0.001, p = 0.001, and p = 0.019, respectively). Further studies are needed worldwide to assess the QoL following the diagnosis of gynecomastia with better data collection tools.
